# Biomechanical characteristics of swing techniques using different clubs in college male golfers

**DOI:** 10.1371/journal.pone.0331051

**Published:** 2025-09-08

**Authors:** Heng Liu, Zhenjun Li, Hongyu Zhou, Zilong Zhao, Baohua Liu, Zefeng Wang

**Affiliations:** 1 School of Sports Training, Tianjin University of Sport, Tianjin, China; 2 School of Tourism and Public Administration, Zhuhai College of Science and Technology, Zhuhai, China; 3 School of Social Sports, Tianjin University of Sport, Tianjin, China; 4 Department of Social Sports, Hebei Sport University, Shijiazhuang, China; 5 China Institute of Sport Science, General Administration of Sport of China, Beijing, China; Ningbo University, CHINA

## Abstract

**Background:**

Golf is a sophisticated sport that integrates precision, skillfulness, and strategic thinking, with swing techniques of different clubs exhibiting distinct biomechanical characteristics. This study aims to investigate the biomechanical characteristics of golfers’ full swings with different clubs from kinematic and dynamics perspectives, thereby providing insights for optimizing full swing techniques.

**Methods:**

Ten low-handicap right-handed college male golfers were recruited, and their full swing parameters with the driver, 5-iron, and 7-iron (each club was successfully collected 10 times) were synchronously collected using a 250 Hz infrared motion capture system and a 1000 Hz three-dimensional force platform. A one-way ANOVA was conducted to compare biomechanical indicators during the swing motion across different clubs.

**Results:**

There were significant kinematic differences between the driver and irons, yet smaller differences between 5-iron and 7-iron. As a whole, irons showed a faster peak time and a smaller angular velocity. The GRF of different clubs exhibited different dynamic characteristics at various swing moments, yet the dynamic regularity remained consistent throughout the full swing. During the downswing, the horizontal angular impulse of the driver was greater than irons, and the frontal angular impulse of the 5-iron was greater than that of both driver and 7-iron.

**Conclusions:**

The characteristics of the driver are slow – paced energy accumulation, delayed acceleration, and horizontal sweeping shots; the characteristics of the 5-iron are that the leading arm coordinates with the frontal angular impulse to optimize the shot trajectory; the characteristics of the 7-iron are compact transition and precise shots driven by the torso.

## Introduction

The swing technique, being the core of golf, with its movement smoothness, force-generation efficiency, and swing stability, is a crucial determinant of a golfer’s shot performance and competition results [1,2]. There are variations in physical parameters, such as shaft length, clubface angle, and swing weight, among different types of golf clubs, including driver, 5-iron, and 7-iron. The driver is utilized for long-distance tee shots, the 5-iron for medium-to-long approach shots in the fairway, and the 7-iron for medium-to-short approach shots targeting the green [3–5]. Tactically, the driver prioritizes distance maximization in tee shots, the 5-iron balances mid-long approach range with trajectory control, and the 7-iron emphasizes precision in short-approach scenarios. This functional differentiation shapes their distinct biomechanical demands. Proficient mastery of full-swing techniques with different clubs enables precise control over shot distance, direction, trajectory, and spin, thereby enhancing overall golf performance [6,7]. Conversely, if the full swing technique is not reasonable and fails to meet the technical requirements of different golf clubs, it will not only be difficult to achieve the desired shot effect, but also increase the risk of sports injuries during long-term training [8].

Full-swing technique research typically employs a phase-partitioned analytical framework. The backswing, as the initial stage of the full swing, involves the body rotating in a direction away from the target to accumulate energy, thus establishing a stable swing rhythm and facilitating the transfer of the center of gravity [9]. The transition, serving as the connecting link between the backswing and the downswing, involves the transfer and adjustment of the body’s center of gravity [10]. The downswing is a crucial phase for energy release, during which the energy is concentrated on the clubhead and released at the moment of impact to achieve a relatively high ball speed [11]. A rational kinematic sequence is conducive to the energy transfer during the swing, and numerous studies have shown that the kinematic sequence in the full swing process reaches peak angular velocity successively from proximal to distal [7,12,13]. This consistent pattern—where the pelvis, torso, lead arm (lead arm is the left forearm for right-handed), and club achieve peak angular velocity in that order—forms the basis of efficient force transmission from the lower body to the clubhead. However, while the order of this sequence is well-established, few studies have explored whether the timing varies across different golf clubs, which differ significantly in physical parameters such as shaft length and inertia. Ground reaction force (GRF) reflects the external force interaction between the golfer and the ground, while angular impulse quantifies the rotational effect of these forces on the body segments. Notably, their coordination is pivotal to swing mechanics: GRF generates the torque that drives angular impulse, with their temporal alignment dictating efficient energy transfer through the kinetic chain.

Han has conducted a GRF analysis of the driver, 5 – iron, and pitching wedge, revealing that the peak three – dimensional GRF in the lead foot positively correlates with clubhead speed [14]. In addition, during the swing of the driver and irons, the GRF are mostly associated with the shot effect. The vertical GRF is a key indicator for evaluating clubhead speed, and changes in the lateral GRF can affect the shot angle [13,15,16]. However, there has been no research on the internal differences in GRF during the full swing of the driver, 5-iron, and 7-iron. Angular impulse is often studied in combination with the swing plane. In the research on the swing planes of the driver, 5-iron, and wedge, Cheon has discovered that an excessive angular impulse during the backswing can lead to a reduction in the swing plane angle, and that the angular impulses of the wedge and the driver are similar during the downswing [17]. During the downswing, the greater the horizontal angular impulse, the faster the clubhead speed of the driver. Moreover, the peak frontal angular impulse of the 6-iron was greater than that of the driver [18]. These findings highlight phase-specific associations between GRF and angular impulse, but gaps remain in understanding how their coordination varies by club type-particularly in temporal synchronization, such as the timing of GRF peaks relative to angular impulse accumulation, and their combined influence on shot outcomes. Grasping the angular impulse characteristics of different clubs is beneficial for enhancing golfers’ performance.

At present, the research landscape exhibits notable lacunae. There is a lack of in-depth exploration into the precise kinematic and dynamics principles governing the driver, 5-iron, and 7-iron. Additionally, the variances among these different golf clubs in terms of kinematics and dynamics remain inadequately studied. In this study, the kinematic characteristics of different clubs were analyzed in terms of the peak angular velocities and peak time to peak values of the pelvis, torso, lead arm, and club across different swing phases. Mechanistically, we hypothesize that longer clubs (driver) delay distal phase initiation to manage higher inertia, while shorter irons (7-iron) accelerate distal timing for precision control. The dynamics characteristics of different clubs were uncovered by examining the ground reaction forces and angular impulses at different moments during the swing.

## Materials and methods

### Participants

Ten male college golfers (all right-handed, mean age = 20.70 ± 1.27, mean experience year = 6.20 ± 3.25, mean handicap = 1.29 ± 1.93) volunteered to participate in the study. Inclusion criteria: No major injuries in the past six months; a practice frequency of at least four times per week; being in good physical condition during the testing period (mean height = 1.77 ± 0.07, mean body mass = 72.65 ± 7.42); no strenuous exercise within 24 hours before the experiment to ensure a stable physical state. All participants signed informed consent forms. This study was approved by the Ethics Committee of Tianjin University of Sport (TJUS2024−062). The recruitment period for this study is from December 30, 2024 to January 20, 2025.

### Equipment and instrumentation

Participants prepared clubs and equipment that complied with the rules of golf competitions on their own. The driver clubhead was made of titanium alloy and the shaft was made of carbon fiber. The clubhead of the iron clubs was made of soft iron and the shaft was made of stainless steel. Twelve Bridgestone - e12 balls were selected as the test balls.

Infrared motion capture system: A Qualisys motion capture system (Qualisys Track Manager, Sweden) equipped with 16 high-speed cameras was used. The sampling frequency was set at 250 Hz. High-speed infrared cameras were employed to capture passive reflective markers, collecting kinematic parameters such as angular velocity and the time to reach peak angular velocity of the pelvis, torso, arms, and clubs during the swings of participants using different clubs.

Force plate: A Kistler force plate (9287C, 900 mm × 600 mm × 100 mm, KISTLER, Switzerland) with a sampling frequency of 1000 Hz was selected. An external amplifier was used to collect ground reaction force data (including vertical, anterior-posterior (AP), and lateral directions) during the swing. The force plate was connected and synchronized with the Qualisys motion capture system.

### Procedure

Before the experiment, a hitting cage with dimensions of 3.15m × 1.6m × 2.8m was set up in the laboratory environment. A hitting mat was laid at a distance of at least 4.5m from the hitting cage. A T-shaped rod was used for field scanning, and an L-shaped rod was used to establish a virtual experimental coordinate system for three-dimensional space calibration [13,19]. Suggested settings based on VIusal3D software operation were X-axis for left-right direction (+ toward the target), Y-axis for front-back direction (+ toward the body), and Z-axis for vertical direction (+ upward).

Warm up: Participants wore tight sportswear pants, were bare-chested, and brought their own golf equipment, gloves, and golf shoes that conformed to golf rules. They carried out a 10 minutes warm up exercise, including dynamic stretching, swing practice, and hitting practice, to adapt to the testing environment, prevent injuries, and improve the testing effect.

Marker point attachment: Based on the Qualisys Sports Marker Set-golf model in the Qualisys system, 16 mm infrared passive reflective markers were used. A total of 35 markers were attached to the human body, and 6 markers were attached to the golf club(see S1 Figa). Ensure that the Marker points were firmly attached to the participants. Participants were required to be familiar with the test items and procedures in advance to avoid interference with their swings.

Swing test: Participants were required to stand on the hitting mat laid with a force plate. After the experimenter observed that the participants were in the ready position, a command was given to start the motion collection. Participants conducted swing hitting tests according to their own swing rhythms. The experimenters stopped the collection after the hitting was completed. First, the swing data of the 7-iron were collected, followed by the 5-iron, and finally the driver. Increasing the test intensity step by step was beneficial to the test results [20]. Ten valid swing data sets were collected for each club. Swing success criteria: the carry distance was within ±10% of the participant’s average distance for that club, and the direction deviated by less than 5° from the target line. The GCQuad high-speed camera sensor (Foresight Sports, USA) monitoring was used to monitor the effectiveness of the participant’s hitting. The interval between each swing was 30 seconds, and the interval between the tests of each club was 3 minutes.

### Operational definitions

Backswing: Begins at TA, defined as the moment when the club speed reaches 0.1 mph. Terminates at TOP, marked by the club’s angular velocity reaching 0 (indicating a directional change) and the maximum posterior rotation of the club relative to the body. Transition: Begins when the angular velocity of any of the pelvis, torso, or lead arm first reaches 0. Ends when the club’s angular velocity reaches 0 and it begins to accelerate downward, marking the transition from backswing to downswing. Downswing: Commences after the end of the transition phase. Terminates at IMP, defined as one frame before the club head hits the ball. The first peak angular velocity refers to the instantaneous angular velocity at which each segment reaches the maximum value of clockwise rotation during the backswing. The second peak angular velocity refers to the instantaneous angular velocity at which each component reaches the maximum value of counterclockwise rotation during the downswing.

GRF: It refers to the force exerted by the ground on the human body when the human body is in contact with the ground. According to Newton’s third law, when the human body exerts a force on the ground, the ground will simultaneously generate a reaction force that is equal in magnitude and opposite in direction. The changes in the three component forces at six swing moments are used to reflect the instantaneous changes in the GRF during the swing.

Angular impulse: It is the integral of the torque with respect to time, which reflects the cumulative effect of the torque over time and describes the change in the angular momentum of an object. The horizontal angular impulse is the integral of the sum of the horizontal torques, reflecting the dynamics effect of the human body’s rotation around the Z-axis. The frontal angular impulse is the integral of the sum of the frontal torques, reflecting the dynamics effect of the human body’s rotation around the Y-axis (see S1 Figb).


$$\begin{array}{c} J=\int_{t0}^{t1}Mdt \end{array}$$ (1)

In the formula: M is the torque, t0 and t1 are the starting time and ending time of the integral respectively, $\int_{t0}^{t1}dt$ is t0 to t1 integral calculation.

### Data analysis

**Fig 1:**
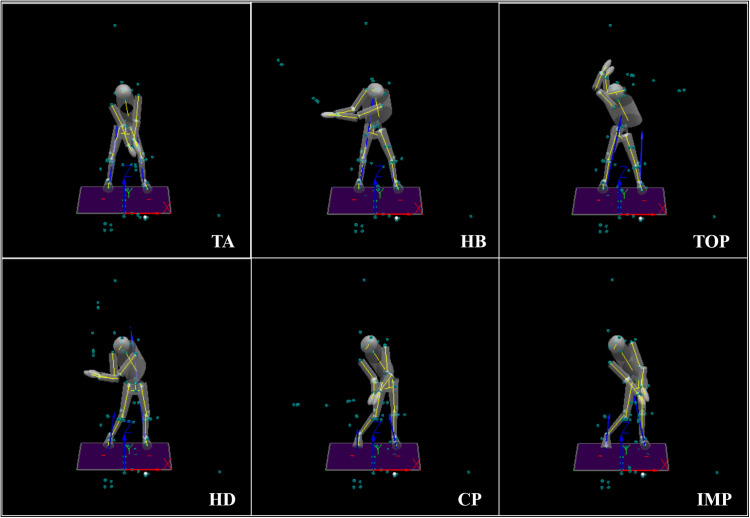
The moments of full swing. TA, the club speed reaches 0.1 mph; HB, the lead arm moves clockwise to the horizontal plane (virtual coordinate); TOP, the angular velocity of the club is 0; HD, the lead arm moves counterclockwise to the horizontal plane; CP, the club moves counterclockwise to the horizontal plane; IMP, one frame before the club head hits the ball.

After the experiment, pre-processing such as motion truncation and point supplementation was carried out on the collected raw data, which was then output in C3D format. The Golf model in Visual3D (C-motion Inc, USA) software was used for filtering, interpolation, and the calculation and processing of biomechanical indices. Motion capture data was filtered using a bi-directional Butterworth low-pass filter with a cutoff frequency of 6 Hz and aligned using timestamps. Based on the technical requirements of the full golf swing and biomechanical principles, this study divided the swing motion into three stages: the backswing stage, the transition stage, and the downswing stage for the study of kinematic characteristics. Moreover, the swing moments were divided for the study of dynamic characteristics (see Fig 1).

### Statistical analysis

The processed biomechanical data were summarized and integrated through Excel 2019 (Microsoft, USA). The valid data of each club for each person were collected. The S-W normality test was conducted using SPSS 27.0 (IBM, USA) software. After inspection, the data conformed to the normal distribution, and a one-way ANOVA was used. The kinematic and dynamic differences under the full-swing techniques of different clubs were compared. Significant difference was set to *p* < 0.05 for all analyses, and effect sizes were calculated using partial eta squared ($\eta_{p}^{2}$). The sample size in each group was equal, and Tukey’s Honestly Significant Difference (HSD) was applied for post-hoc analysis.

## Results

### Backswing phase kinematics

Table 1 shows that the time of first peak angular velocity and the first peak angular velocity. Time of first peak angular velocity: A one-way ANOVA revealed differences on torso (*p* = 0.001, $\eta_{p}^{2}$ = 0.045) and club (*p* < 0.001, $\eta_{p}^{2}$ = 0.046). A Tukey HSD post-hoc analysis showed that torso during driver backswing longer than 5-iron (*p* = 0.008), and longer than 7-iron (*p* = 0.002); club during driver backswing longer than 5-iron (*p* = 0.007), and longer than 7-iron (*p* = 0.002). There were no differences on pelvis and lead arm (both *p* > 0.05). First peak angular velocity: There were significant differences on pelvic and torso and club (both *p* < 0.05). The driver swing’s pelvic of the first peak angular velocity faster than 5-iron (*p* = 0.012) and faster than 7-iron (*p* = 0.018); the driver swing’s torso of the first peak angular velocity faster than 5-iron (*p* = 0.002) and faster than 7-iron (*p* = 0.001); the driver swing’s club of the first peak angular velocity faster than 5-iron (*p* = 0.024) and faster than 7-iron (*p* = 0.013). There was no difference on lead arm (*p* = 0.089, $\eta_{p}^{2}$ = 0.016).

**Table 1:** Backswing Kinematic Parameters.

Kinematic indices		Driver	5-iron	7-iron	*F*	*p*	$\eta_{p}^{2}$
		*M ± SD*	*M ± SD*	*M ± SD*			
Time of first peak angular velocity (ms)	Pelvis	498.44 ± 194.83	444.48 ± 171.05	453.72 ± 184.10	2.471	0.086	0.016
	Torso	557.80 ± 200.23^a^**	483.60 ± 160.42	473.76 ± 158.98^c^**	6.951	0.001	0.045
	Lead arm	607.04 ± 179.89	563.40 ± 162.19	545.28 ± 150.49^c^*	3.718	0.025	0.024
	Club	693.52 ± 156.47^a^**	629.44 ± 145.74	622.12 ± 140.11^c^**	7.083	<0.001	0.046
First peak angular velocity (°/s)	Pelvis	−135.79 ± 51.12^a^*	−117.76 ± 38.78	−118.55 ± 42.82^c^*	5.324	0.006	0.034
	Torso	−259.52 ± 124.37^a^**	−220.22 ± 49.48	−218.54 ± 47.07^c^**	8.014	<0.001	0.051
	Lead arm	−329.01 ± 82.14	−317.19 ± 54.50	−308.35 ± 59.03	2.442	0.089	0.016
	Club	−627.60 ± 134.64^a^*	−582.96 ± 119.07	−579.17 ± 104.68^c^*	5.030	0.007	0.033

^*a*^, significant differences between driver and 5-iron; ^*c*^, significant differences between driver and 7-iron. *p < 0.05, **p < 0.01.

### Transition phase kinematics

As shown in Table 2, there were significant differences on the time for torso to reach TOP (*p* < 0.001, $\eta_{p}^{2}$ = 0.054) and lead arm (*p* = 0.006, $\eta_{p}^{2}$ = 0.034). The torso during driver transition to reach TOP longer than 5-iron (*p* = 0.001) and longer than 7-iron (*p* = 0.001); the lead arm during driver transition to reach TOP longer than 7-iron (*p* = 0.006). There was no difference on pelvis (*p* = 0.265, $\eta_{p}^{2}$ = 0.009). There were significant differences on the time for pelvic to reach the second peak angular velocity (*p* = 0.001, $\eta_{p}^{2}$ = 0.044) and lead arm (*p* < 0.001, $\eta_{p}^{2}$ = 0.044). The pelvic during driver transition to reach the second peak angular velocity longer than 5-iron (*p* = 0.011) and longer than 7-iron (*p* = 0.002); the lead arm during driver transition to reach the second peak angular velocity longer than 5-iron (*p* = 0.025) and longer than 7-iron (*p* < 0.001). There were no differences on torso and lead arm (both *p* > 0.05).

**Table 2:** Transition Kinematic Parameters.

Kinematic indices		Driver	5-iron	7-iron	*F*	*p*	$\eta_{p}^{2}$
		*M ± SD*	*M ± SD*	*M ± SD*			
Time of TOP (ms)	Pelvis	69.92 ± 36.63	63.04 ± 32.74	63.28 ± 31.86	1.334	0.265	0.009
	Torso	38.86 ± 22.29^a^**	27.54 ± 21.00	27.40 ± 24.64^c^**	8.402	<0.001	0.054
	Lead arm	25.44 ± 41.64	15.54 ± 24.09	12.42 ± 19.34^c^**	5.157	0.006	0.034
	Club	0.00 ± 0.00	0.00 ± 0.00	0.00 ± 0.00	/	/	/
Time of second peak angular velocity (ms)	Pelvis	252.84 ± 34.89^a^*	237.32 ± 39.67	234.52 ± 39.13^c^**	6.760	0.001	0.044
	Torso	231.94 ± 39.46	220.74 ± 46.99	222.32 ± 49.73	1.768	0.173	0.012
	Lead arm	236.92 ± 58.00^a^*	221.22 ± 35.57	213.36 ± 27.14^c^**	8.045	<0.001	0.051
	Club	268.60 ± 28.29	260.76 ± 26.21	260.28 ± 27.63	2.908	0.056	0.019

TOP defined as a club with an angular velocity of 0. Club indices no statistically significant in the transition. ^*a*^, significant differences between driver and 5-iron; ^*c*^, significant differences between driver and 7-iron. *p < 0.05, **p < 0.01.

### Downswing phase kinematics

Table 3 shows that the time of second peak angular velocity and the second peak angular velocity. Time of second peak angular velocity: There were significant differences on pelvic (*p* = 0.010, $\eta_{p}^{2}$ = 0.031), lead arm (*p* = 0.015, $\eta_{p}^{2}$ = 0.028) and club (*p* = 0.173, $\eta_{p}^{2}$ = 0.012). The pelvic during driver downswing longer than 5-iron (*p* = 0.021), and longer than 7-iron (*p* = 0.024); the lead arm during driver downswing longer than 7-iron (*p* = 0.011); the club during driver downswing longer than 7-iron (*p* = 0.018). There was no difference on torso (*p* = 0.939, $\eta_{p}^{2}$ = 0.000). Second peak angular velocity: There were significant differences on pelvic (P = 0.003, $\eta_{p}^{2}$ = 0.039), torso (*p* < 0.001, $\eta_{p}^{2}$ = 0.087) and lead arm (*p* < 0.001, $\eta_{p}^{2}$ = 0.071). The driver swing’s pelvic of the second peak angular velocity faster than 5-iron (*p* = 0.039) and faster than 7-iron (*p* = 0.002); the driver swing’s torso of the second peak angular velocity faster than 5-iron (*p* < 0.001) and faster than 7-iron (*p* < 0.001); the driver swing’s lead arm of the second peak angular velocity faster than 5-iron (*p* = 0.010) and faster than 7-iron (*p* < 0.001). There was no difference on club (*p* = 0.574, $\eta_{p}^{2}$ = 0.004) but 7-iron (2095.86 ± 239.30 °/s) faster than 5-iron (2092.44 ± 174.86 °/s).

**Table 3:** Downswing Kinematic Parameters.

Kinematic indices		Driver	5-iron	7-iron	*F*	*p*	$\eta_{p}^{2}$
*M ± SD*	*M ± SD*	*M ± SD*
Time of second peak angular velocity (ms)	Pelvis	187.12 ± 55.40^a^*	170.44 ± 40.14	170.76 ± 33.10^c^*	4.727	0.010	0.031
	Torso	196.88 ± 52.79	194.68 ± 41.14	195.16 ± 43.84	0.063	0.939	0.000
	Lead arm	215.48 ± 53.52	206.68 ± 30.83	198.48 ± 35.82^c^*	4.253	0.015	0.028
	Club	272.08 ± 47.25	260.40 ± 26.25	263.29 ± 38.29^c^*	4.191	0.016	0.027
Second peak angular velocity (°/s)	Pelvis	515.21 ± 78.90^a^*	485.56 ± 78.87	474.41 ± 97.64^c^**	6.069	0.003	0.039
	Torso	759.52 ± 90.62^a^**	705.09 ± 81.47	692.75 ± 108.56^c^**	14.216	<0.001	0.087
	Lead arm	1138.32 ± 140.52^a^**	1065.97 ± 119.23	1045.15 ± 171.59^c^**	11.315	<0.001	0.071
	Club	2119.84 ± 179.73	2092.44 ± 174.86	2095.86 ± 239.30	0.557	0.574	0.004

^*a*^, significant differences between driver and 5-iron; ^*c*^, significant differences between driver and 7-iron. *p < 0.05, **p < 0.01.

### Ground reaction force analysis

**Fig 2:**
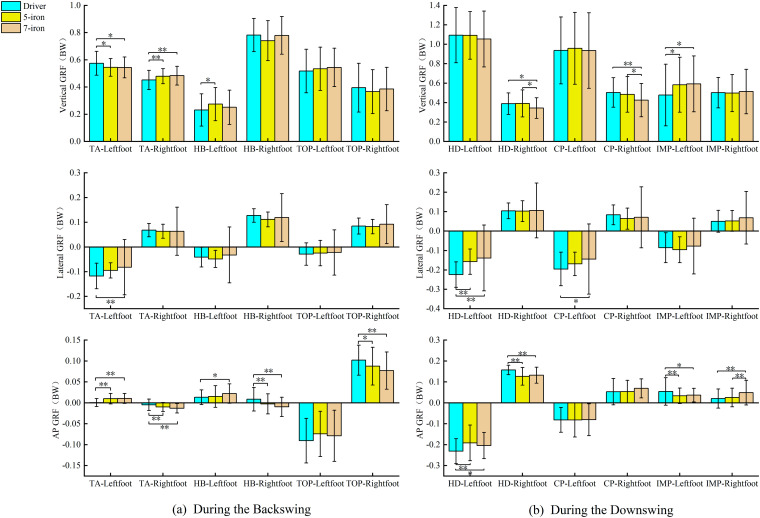
GRF at different swing moments. (a) The GRF at swing moments during the backswing;(b) The GRF at swing moments during the downswing. *p < 0.05, **p < 0.01.

During the backswing, as shown in Fig 2a that the GRF of both feet at TA, HB and TOP. There were significant differences on the vertical GRF of left foot at TA (*p* = 0.006, $\eta_{p}^{2}$ = 0.034), the right foot at TA (*p* = 0.001, $\eta_{p}^{2}$ = 0.045) and the left foot at HB (*p* = 0.045, $\eta_{p}^{2}$ = 0.021). Regarding the vertical GRF near HB, the right foot reached its maximum, and the left foot dropped to its minimum. There was significant difference on the lateral GRF of left foot at TA (*p* = 0.003, $\eta_{p}^{2}$ = 0.038). The driver lateral GRF of left foot at TA less than 7-iron (*p* = 0.002). The lateral GRF of both feet continuously increased in the direction of the target. There were significant differences on the AP GRF of left foot at TA, the right foot at TA, the left foot at HB, the right foot at HB and the right foot at TOP (all *p* < 0.05). The AP GRF reached 0 BW% near HB. The right foot reached its maximum before HB, and the left foot dropped to its minimum.

During the downswing, as shown in Fig 2b that the GRF of both feet at HD, CP and IMP. There were significant differences on the vertical GRF of right foot at HD, (*p* = 0.007, $\eta_{p}^{2}$ = 0.033), the right foot at CP (*p* = 0.003, $\eta_{p}^{2}$ = 0.038) and the left foot at IMP (*p* = 0.010, $\eta_{p}^{2}$ = 0.030). The vertical GRF of the left foot reached its maximum near HD. The vertical GRFs of both feet dropped to their minimum values from CP to IMP. There were significant differences on the lateral GRF of left foot at HD, (*p* < 0.001, $\eta_{p}^{2}$ = 0.098) and the left foot at CP (*p* = 0.012, $\eta_{p}^{2}$ = 0.029). The lateral GRF of both feet reached their maximum from TOP to HD, and dropped to their minimum from HD to IMP. There were significant differences on the AP GRF of left foot at HD, the right foot at HD, the left foot at IMP and the right foot at IMP (all *p* < 0.01). The AP GRF of the left foot dropped to its minimum from TOP to HD, and the AP GRF of the right foot reached its maximum from TOP to CP.

### Angular impulse analysis

**Fig 3:**
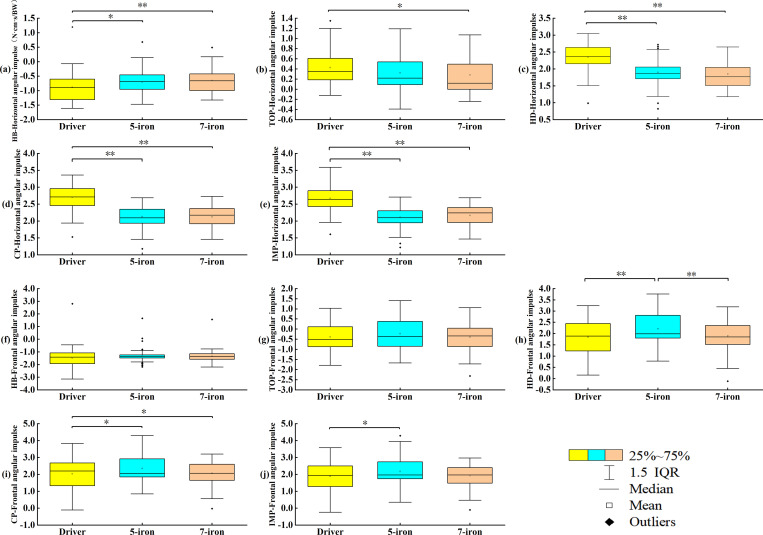
Angular impulse at different swing moments. (a-e) Horizontal angular impulse at different swinging moments; (f-j) Frontal angular impulse at different swinging moments. *p < 0.05, **p < 0.01.

Fig 3a–3e shows the horizontal angular impulse during the swing with different clubs. There were significant differences on the horizontal angular impulse at HB, at TOP, at HD, at CP and at IMP (*p* < 0.05). The horizontal angular impulse of driver at HB less than 5-iron (*p* = 0.034) and less than 7-iron (*p* = 0.002); the horizontal angular impulse of driver at TOP greater than 7-iron (*p* = 0.019); the horizontal angular impulse of driver at HD greater than 5-iron (*p* < 0.001) and greater than 7-iron (*p* < 0.001); the horizontal angular impulse of driver at CP greater than 5-iron (*p* < 0.001) and greater than 7-iron (*p* < 0.001); the horizontal angular impulse of driver at CP greater than 5-iron (*p* < 0.001) and greater than 7-iron (*p* < 0.001); the horizontal angular impulse of driver at IMP greater than 5-iron (*p* < 0.001) and greater than 7-iron (*p* < 0.001). Fig 3f–3j shows the frontal angular impulse during the swing with different clubs. There were significant differences on the frontal angular impulse at HD (*p* < 0.001, $\eta_{p}^{2}$ = 0.050), at CP, (*p* = 0.006, $\eta_{p}^{2}$ = 0.034), at IMP (*p* = 0.019, $\eta_{p}^{2}$ = 0.026). The frontal angular impulse of 5-iron at HD greater than driver (*p* < 0.001) and greater than 7-iron (*p* = 0.006); the frontal angular impulse of diver at CP less than 5-iron (*p* = 0.011) and less than 7-iron (*p* = 0.024); the frontal angular impulse of diver at IMP less than 5-iron (*p* = 0.024).

## Discussion

This study investigated the characteristics of the swing techniques of different clubs through the means of sports biomechanics, and compared the differences from kinematics and dynamics during swing. The study found that while drivers and irons had different kinematic characteristics yet all clubs showed consistent GRF, with driver accumulating more horizontal angular impulse and 5-iron accumulating more frontal angular impulse, thus helping researchers better understand the biomechanical characteristics of different clubs.

### Kinematic characteristics

**Fig 4:**
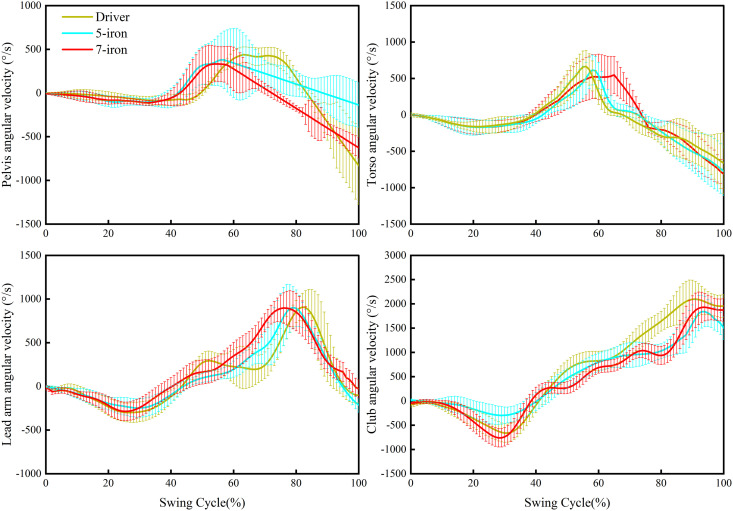
Time series of Pelvis, Torso, Lead arm and Club angular velocities. Swing Cycle is the TA to IMP normalized time.

The sequence in each segment of different clubs reached the first peak angular velocity was from the proximal end to the distal end, which was consistent with previous research [7,12]. The driver had the longest shaft, so its power-chain activation time extended by 18–22 ms compared to irons. The increase in moment of inertia from the club length was compensated by increasing pelvic rotation angle [21,22]. To address the prolonged pelvic rotation time, separation rotation training such as seated medicine ball turn-and-throw drills should be incorporated to enhance pelvic-thoracic rotational separation [23]. Specifically, perform 3 sets of 12 repetitions (6 per side) with 60 seconds of rest between sets. This exercise emphasizes prioritizing pelvic rotation while stabilizing the torso until force exertion, to refine timing coordination between pelvic and thoracic movements. The longer shaft also generated a greater centrifugal force. The resultant higher centrifugal forces observed with the driver may impose greater demands on trunk stabilization mechanisms as described in studies linking rotational kinetics to core muscle function [24,25]. Future work incorporating electromyography (EMG) would be essential to directly quantify muscle activation patterns and contraction types under these specific force conditions. The kinematic sequence of each segment’s 0 °/s for different clubs was from proximal to distal. During the transition, the driver’s torso 0 °/s occurred significantly earlier than that of the 5-iron and 7-iron. Premature spine braking at 0 °/s increased lumbar muscle pressure, and long-term intense training could cause lumbar muscle strain [26]. It led to an increase in the peak tension of the erector spinae muscles, and long-term training was likely to cause degeneration of the L4-L5 intervertebral disc [27]. The 5-iron balanced the demands for power and control by optimizing the timing of humeral internal rotation [28–30]. This study found that irons prioritized precise control over peak maximization, with the lead arm reaching the second peak angular velocity earlier before IMP as the club shaft got shorter. During the downswing, each segment of the driver took longer overall than irons, allowing it to achieve a greater peak angular velocity. The driver maximized club speed by extending the acceleration time. The Fig 4 reveal nuanced dynamic patterns of the pelvis, torso, lead arm, and club across the swing cycle for Driver, 5-iron, and 7-iron. Pelvic, torso and lead arm angular velocities tended to decrease after 80% of the swing cycle. Club angular velocity was maintained after peaking prior to impact.

The proprioceptive system monitored muscle tension in real-time through the Golgi tendon organs and adjusted the release timing of the wrist joint [31,32]. This implies that irons rely more on the “whiplash effect” to enhance the shotting efficiency. To optimize wrist control during impact and enhance whip-like efficiency, delayed release drills such as resistance band simulations of the downswing are recommended [33]. Use a medium-resistance band anchored at waist height, performing 4 sets of 8 repetitions with 45 seconds of rest between sets. Each repetition begins from the backswing top, with controlled downswing initiation and deliberate delay of wrist release until near the impact phase, focusing on stabilizing wrist angles to improve precision.

### Dynamic characteristics

**Fig 5:**
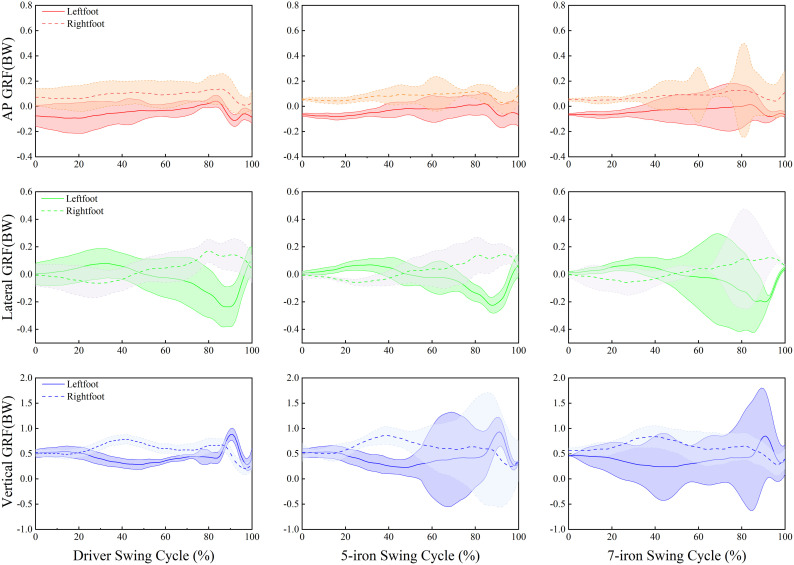
Time series of GRF. Swing Cycle is the TA to IMP normalized time.

GRF could clearly reflect the force exerting situation in each phase of the swing and played an important role in the construction of the power chain [34]. There is a consistency in the trend of GRF across clubs in the swing cycle (Fig 5). During the first 80% of the swing cycle, the AP GRF of both feet showed consistent trends; in contrast, the lateral GRF crossed and exhibited opposite trends around the 40–50% swing cycle, while the vertical GRF displayed reversed patterns through crossing at the 80–90% swing cycle.

**Fig 6:**
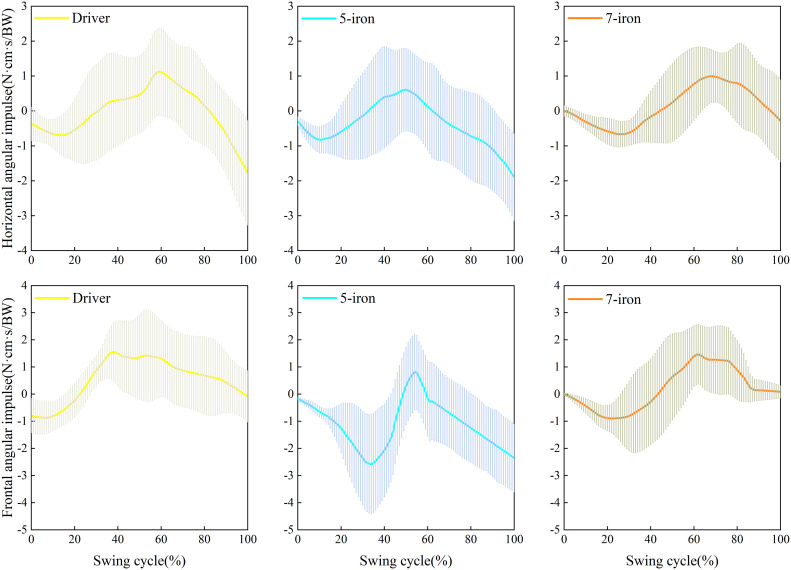
Time series of angular impulse. Swing Cycle is the TA to IMP normalized time.

During the swing of different clubs, the GRF shows similar characteristics (Fig 2). The vertical GRF of the right foot reached the highest, while that of the left foot dropped to the lowest [35]. At HB, the golfers generated an upward reaction force by pushing off the ground with the right foot, providing power for the rotation of the upper body and the swing. When using the driver at TOP, golfers were more inclined to shift their center of gravity to the left foot in advance, generating greater swing torque. An early increase in the vertical GRF of the left foot maybe exerted greater pressure on the left ankle, knee, and pelvic, resulting in cartilage wear and ligament strains in these joints. At TA, the lateral GRF of the left foot of the driver was significantly less than 7-iron, and the center of gravity tended to the right side [36]. Xu et al found that adjusting the ankle initial contact angle (AICA) to the 30°-40° range significantly reduced peak vertical ground reaction force (PVGRF) and anterior cruciate ligament (ACL) loading, a mechanism that has direct reference to the prevention of lower extremity injuries triggered by an early surge in GRF in the golf swing [37]. During the downswing of the driver, the left foot needed to brake in a direction away from the target to facilitate the club face’s return to a square position and improve the accuracy of the shot. The AP GRF of the left and right feet showed dynamic changes at TA, which was the main cause of the formation of the backswing plane. The swing width in the backswing could be increased more effectively through the changes in the anteroposterior force of the right foot [38,39]. This study found that at IMP, the AP GRF of the right foot of the 7-iron was significantly greater than driver and 5-iron with its right foot applying more force to the ground, thus facilitating the downward attack angle. Horizontal and frontal angle pulses exhibited different club-specific and phase-dependent patterns throughout the swing cycle (Fig 6).

Horizontal angle impulse peaks at 40−60% of the swing cycle for the 5-iron and 60−80% of the swing cycle for the driver and 7-iron. 5-iron frontal angle impulse is minimized during the 20−40% swing cycle. The horizontal angular impulse reflected the club speeds and trajectories of different clubs. Longer club shafts had greater inertia during the swing [40,41]. At TA, golfers applied more force in the direction away from the target to initiate the swing. During the downswing, the driver continuously accumulated horizontal angular impulse, which reflected its unique mechanical characteristics (Fig 3c–3e). However, due to continuous twisting and force exertion, the waist was at an increased risk of lumbar degenerative diseases [8,26,42]. While the current kinetic data identifies this loading pattern, EMG validation in future studies is required to elucidate the specific muscle activation strategies (e.g., core bracing, eccentric control) employed to manage these forces. This study found that the continuously increasing horizontal angular impulse of the driver enabled efficient energy transfer, while the irons achieved accuracy through timing control. The frontal angular impulse reflected the shot angles and swing planes of different clubs. During the backswing, there was no difference in the frontal angular impulse among different clubs, and this phase was governed by common biomechanical principles (Fig 3f–3g). At HD, when using 5-iron, golfers needed to apply a greater frontal angular impulse to keep the lead arm ahead of the club. If the way of exerting force was incorrect, the meniscus bore a great amount of shear force, resulting in injury and affecting the motor function of the knee joint [43]. A smaller frontal angular impulse could prevent the driver from attacking too much downward and hitting the ball with a sweeping motion. While the larger frontal angular impulse of the 5-iron at IMP could generate more downward shot angles, which was conducive to precisely controlling the landing point.

### Limitations and prospects

Only ten low handicap, righthanded male college golfers were recruited, ignoring the differences in gender, age, skill levels, and physical characteristics. Sequential effects were neglected in this study by using fixed club sequential measurements. Randomized measurements were taken in future studies to avoid the associated risks. The characteristics of muscle strength were also important biomechanical features, yet the instantaneous changes in the force exerted by the muscles had not been investigated. The influence of the complex actual course environment on the biomechanical characteristics of swing techniques was also not considered. Discrete parameter-based analysis can reveal the biomechanical characteristics of different clubs at critical moments of the swing technique, but for future studies, statistical parameter mapping (SPM) analysis should be used to more sensitively detect kinematic differences at specific stages throughout the swing cycle [44]. In the future, mathematical modeling can be applied to comprehensively consider the interrelationships among various factors such as surface electromyography, shot effect, the environment, and clubs. Future work should integrate the data-driven ligament fatigue framework to quantify cumulative damage in lumbar discs during repetitive swings, as its hyperelastic-viscoelastic constitutive model overcomes static-loading limitations in predicting degeneration risks from angular impulse-induced torsional stresses [45].

## Conclusions

The driver has a slow backswing to accumulate potential energy, and in downswing, it achieves a long hitting distance through specific means. When training the driver, focus on lengthening the acceleration time of the downswing phase, and strengthen the timing of the pelvis and torso to enhance the efficiency of energy transfer. The 5-iron has a moderate backswing rhythm, and the peak value of the vertical GRF of the right foot optimizes the hitting angle at IMP. When practicing the 5-iron, focus on the peak control of the vertical GRF of the right foot, optimizing the angle of the stroke by adjusting the timing of the weight shift, and balancing power and accuracy. The 7-iron has a fast backswing, with a high AP GRF of the right foot at IMP, and the transition between each segment is compact. Training on the 7-iron swing, emphasizing a fast and consistent swing tempo, improving the tightness of the links and ensuring precise ball control. In conclusion, the driver 5-iron and 7-iron exhibit distinct kinematic and dynamics characteristics. Golfers need to consider factors such as hitting requirements and physical tolerance, and then rationally select clubs and optimize their technical movements accordingly.

## Supporting information

S1 Fig (a) Maker Placement Diagram; (b) Local Segment Coordinate System. (TIF)

## References

[1] Brožka M, Kotrba M, Zahálka F, Gryc T. Impact factors analysis: Differences in high-level golfers. International Journal of Performance Analysis in Sport. 2023;23(5):400–12. doi: 10.1080/24748668.2023.2238168

[2] James N. Performance analysis of golf: Reflections on the past and a vision of the future. International Journal of Performance Analysis in Sport. 2009;9(2):188–209. doi: 10.1080/24748668.2009.11868476

[3] Yang C-C, Chang C-C, Chao T, Tai H-L, Tsai Y-S. The effects of different iron shaft weights on golf swing performance. Front Bioeng Biotechnol. 2024;12:1343530. doi: 10.3389/fbioe.2024.1343530 38380262 PMC10877370

[4] Shan G, Zhang X, Li X, Hao W, Witte K. Quantification of Golfer-club Interaction and Club-type’s Affect on Dynamic Balance during a Golf Swing. International Journal of Performance Analysis in Sport. 2011;11(3):417–26. doi: 10.1080/24748668.2011.11868561

[5] Corke TW, Betzler NF, Wallace ES, Otto SR. Predicting golf ball launch characteristics using iron clubhead presentation variables and the influence of mishits. Proceedings of the Institution of Mechanical Engineers, Part P: Journal of Sports Engineering and Technology. 2021;236(2):124–33. doi: 10.1177/1754337120987857

[6] Hasegawa Y, Okada A, Fujii K. Putting performance bias to the front-lower side of the hole on steep slopes: Differences in strategies and movements between professional and amateur golfers. PLoS ONE. 2024;19(12):e0314820. doi: 10.1371/journal.pone.0314820 PMC11687870 39739896

[7] Tinmark F, Hellström J, Halvorsen K, Thorstensson A. Elite golfers’ kinematic sequence in full-swing and partial-swing shots. Sports Biomech. 2010;9(4):236–44. doi: 10.1080/14763141.2010.535842 21309298

[8] Skibski A, Burkholder E, Goetschius J. Transverse abdominis activity and ultrasound biofeedback in college golfers with and without low back pain. Phys Ther Sport. 2020;46:249–53. doi: 10.1016/j.ptsp.2020.10.004 33059233

[9] Gould ZI, Oliver JL, Lloyd RS, Neil R, Bull M. The Golf Movement Screen Is Related to Spine Control and X-Factor of the Golf Swing in Low Handicap Golfers. J Strength Cond Res. 2021;35(1):240–6. doi: 10.1519/JSC.0000000000002664 29979282

[10] Pan X, Soh KG, Jaafar WMW, Soh KL, Deng N, Cao S, et al. Mental fatigue in golf: A systematic review. PLoS One. 2025;20(2):e0310403. doi: 10.1371/journal.pone.0310403 39977446 PMC11841881

[11] Li B, Wang J, Wu C, Hu Z, Li J, Nam S-C, et al. Effects of Ground Slopes on Erector Spinae Muscle Activities and Characteristics of Golf Swing. Int J Environ Res Public Health. 2023;20(2):1176. doi: 10.3390/ijerph20021176 36673931 PMC9858818

[12] Teu KK, Kim W, Fuss FK, Tan J. The analysis of golf swing as a kinematic chain using dual Euler angle algorithm. J Biomech. 2006;39(7):1227–38. doi: 10.1016/j.jbiomech.2005.03.013 15936026

[13] Ball K, Best R. Centre of pressure patterns in the golf swing: individual-based analysis. Sports Biomech. 2012;11(2):175–89. doi: 10.1080/14763141.2012.673007 22900399

[14] Han KH, Como C, Kim J, Lee S, Kim J, Kim DK, et al. Effects of the golfer-ground interaction on clubhead speed in skilled male golfers. Sports Biomech. 2019;18(2):115–34. doi: 10.1080/14763141.2019.1586983 31042142

[15] Smith AC, Roberts JR, Kong PW, Forrester SE. Comparison of centre of gravity and centre of pressure patterns in the golf swing. Eur J Sport Sci. 2017;17(2):168–78. doi: 10.1080/17461391.2016.1240238 27737623

[16] Jones KM, Wallace ES, Otto SR. Differences in the structure of variability in ground reaction force trajectories provide additional information about variability in the golf swing. Proceedings of the Institution of Mechanical Engineers, Part P: Journal of Sports Engineering and Technology. 2018;232(4):375–84. doi: 10.1177/1754337118772418

[17] Cheon M, Khuyagbaatar B, Yeom J-H, Kim YH. Analysis of swing tempo, swing rhythm, and functional swing plane slope in golf with a wearable inertial measurement unit sensor. J Mech Sci Technol. 2020;34(7):3095–101. doi: 10.1007/s12206-020-0640-3

[18] Peterson TJ, Wilcox RR, McNitt-Gray JL. Angular Impulse and Balance Regulation During the Golf Swing. J Appl Biomech. 2016;32(4):342–9. doi: 10.1123/jab.2015-0131 26958870

[19] Foxworth JL, Millar AL, Long BL, Way M, Vellucci MW, Vogler JD. Hip joint torques during the golf swing of young and senior healthy males. J Orthop Sports Phys Ther. 2013;43(9):660–5. doi: 10.2519/jospt.2013.4417 23886577

[20] Morrison A, McGrath D, Wallace ES. The relationship between the golf swing plane and ball impact characteristics using trajectory ellipse fitting. J Sports Sci. 2018;36(3):303–10. doi: 10.1080/02640414.2017.1303187 28294698

[21] Kim TH, Jagacinski RJ, Lavender SA. Age-related differences in the rhythmic structure of the golf swing. J Mot Behav. 2011;43(6):433–44. doi: 10.1080/00222895.2011.624565 22004259

[22] MacKenzie SJ, Boucher DE. The influence of golf shaft stiffness on grip and clubhead kinematics. J Sports Sci. 2017;35(2):105–11. doi: 10.1080/02640414.2016.1157262 26967490

[23] Coughlan D, Taylor MJD, Jackson J, Ward N, Beardsley C. Physical Characteristics of Youth Elite Golfers and Their Relationship With Driver Clubhead Speed. J Strength Cond Res. 2020;34(1):212–7. doi: 10.1519/JSC.0000000000002300 29065053

[24] Lephart SM, Smoliga JM, Myers JB, Sell TC, Tsai Y-S. An eight-week golf-specific exercise program improves physical characteristics, swing mechanics, and golf performance in recreational golfers. J Strength Cond Res. 2007;21(3):860–9. doi: 10.1519/R-20606.1 17685707

[25] Zou Y, MacFarlane N. Influence of biceps-triceps ratio on golf swing performance. PLoS One. 2024;19(7):e0307547. doi: 10.1371/journal.pone.0307547 39042614 PMC11265706

[26] Lim Y-T, Chow JW, Chae W-S. Lumbar spinal loads and muscle activity during a golf swing. Sports Biomech. 2012;11(2):197–211. doi: 10.1080/14763141.2012.670662 22900401

[27] Parker J, Lagerhem C, Hellström J, Olsson MC. Effects of nine weeks isokinetic training on power, golf kinematics, and driver performance in pre-elite golfers. BMC Sports Sci Med Rehabil. 2017;9:21. doi: 10.1186/s13102-017-0086-9 29238597 PMC5725976

[28] Faux L, Carlisle A, Vickers J, Diss C. The effect of alterations in foot centre of pressure on lower body kinematics during the five-iron golf swing. J Sports Sci. 2019;37(17):2014–20. doi: 10.1080/02640414.2019.1614714 31076017

[29] Fedorcik GG, Queen RM, Abbey AN, Moorman CT 3rd, Ruch DS. Differences in wrist mechanics during the golf swing based on golf handicap. J Sci Med Sport. 2012;15(3):250–4. doi: 10.1016/j.jsams.2011.10.006 22154489

[30] Park T-J, Seo K-E. A Comparative Analysis of X-factor Stretch between Driver and Iron Swing in Male Professional Golfers. Korean Journal of Sport Biomechanics. 2010;20(4):487–95. doi: 10.5103/kjsb.2010.20.4.487

[31] Jagacinski RJ, Kim TH, Lavender SA. Managing the rhythmic complexity of hitting a golf ball. J Mot Behav. 2009;41(5):469–77. doi: 10.3200/35-08-075 19508955

[32] Sorbie GG, Hunter HH, Grace FM, Gu Y, Baker JS, Ugbolue UC. An electromyographic study of the effect of hand grip sizes on forearm muscle activity and golf performance. Res Sports Med. 2016;24(3):222–33. doi: 10.1080/15438627.2016.1191492 27267082

[33] Shaw J, Gould ZI, Oliver JL, Lloyd RS. Twelve Weeks of Progressive Resistance Training Positively Improves Physical Fitness and Golf Swing Performance in Talented Youth Golfers. J Strength Cond Res. 2024;38(6):1103–10. doi: 10.1519/JSC.0000000000004753 38373078

[34] Choi A, Kang TG, Mun JH. Biomechanical evaluation of dynamic balance control ability during golf swing. Journal of Medical Biological Engineering. 2016;36:430–9.

[35] Kim J, Youm C, Son M, Lee M, Kim Y. Golf club characteristics and vertical force distribution associated with pitch and lob shots of different carry distances. International Journal of Sports Science & Coaching. 2017;12(4):540–8. doi: 10.1177/1747954117721268

[36] Kim SE, Lee J, Lee SY, Lee HD, Lee SC, Shim JK. Golf swing in response to anteroposterior ball position. International Journal of Sports Science Coaching. 2023;18(5):1639–48.

[37] Xu D, Zhou H, Quan W, Ma X, Chon T-E, Fernandez J, et al. New Insights Optimize Landing Strategies to Reduce Lower Limb Injury Risk. Cyborg Bionic Syst. 2024;5:0126. doi: 10.34133/cbsystems.0126 38778877 PMC11109754

[38] Navarro E, Mancebo JM, Farazi S, del Olmo M, Luengo D. Foot Insole Pressure Distribution during the Golf Swing in Professionals and Amateur Players. Applied Sciences. 2021;12(1):358. doi: 10.3390/app12010358

[39] Sohn J, Choi H. Are golf-shots distinguished by power control? Or it is just individual differences? International Journal of Performance Analysis in Sport. 2013;13(1):212–24. doi: 10.1080/24748668.2013.11868643

[40] Peterson TJ, McNitt-Gray JL. Regulation of Linear and Angular Impulse during the Golf Swing with Modified Address Positions. J Appl Biomech. 2019;35(1):25–31. doi: 10.1123/jab.2017-0163 30080427

[41] Yeemin W, Kemarat S, Theanthong A. The effects of post activation potentiation warm-up and pre-shot routine programs on driving performance in amateur golfers. PLoS One. 2020;15(10):e0240881. doi: 10.1371/journal.pone.0240881 33079942 PMC7575068

[42] Glofcheskie GO, Brown SHM. Athletic background is related to superior trunk proprioceptive ability, postural control, and neuromuscular responses to sudden perturbations. Hum Mov Sci. 2017;52:74–83. doi: 10.1016/j.humov.2017.01.009 28135584

[43] Hamai S, Miura H, Higaki H, Shimoto T, Matsuda S, Okazaki K, et al. Three-dimensional knee joint kinematics during golf swing and stationary cycling after total knee arthroplasty. J Orthop Res. 2008;26(12):1556–61. doi: 10.1002/jor.20671 18524002

[44] Yona T, Kamel N, Cohen-Eick G, Ovadia I, Fischer A. One-dimension statistical parametric mapping in lower limb biomechanical analysis: A systematic scoping review. Gait Posture. 2024;109:133–46. doi: 10.1016/j.gaitpost.2024.01.018 38306782

[45] Xu D, Zhou H, Jie T, Zhou Z, Yuan Y, Jemni M, et al. Data-driven deep learning for predicting ligament fatigue failure risk mechanisms. International Journal of Mechanical Sciences. 2025;301:110519. doi: 10.1016/j.ijmecsci.2025.110519

