# Supplementary figures and images for: Biomechanical characteristics of swing techniques using different clubs in college male golfers
Source: PLoS One. 2025 Sep 8;20(9):e0331051. doi: 10.1371/journal.pone.0331051 (PMC12416643; doi:10.1371/journal.pone.0331051)

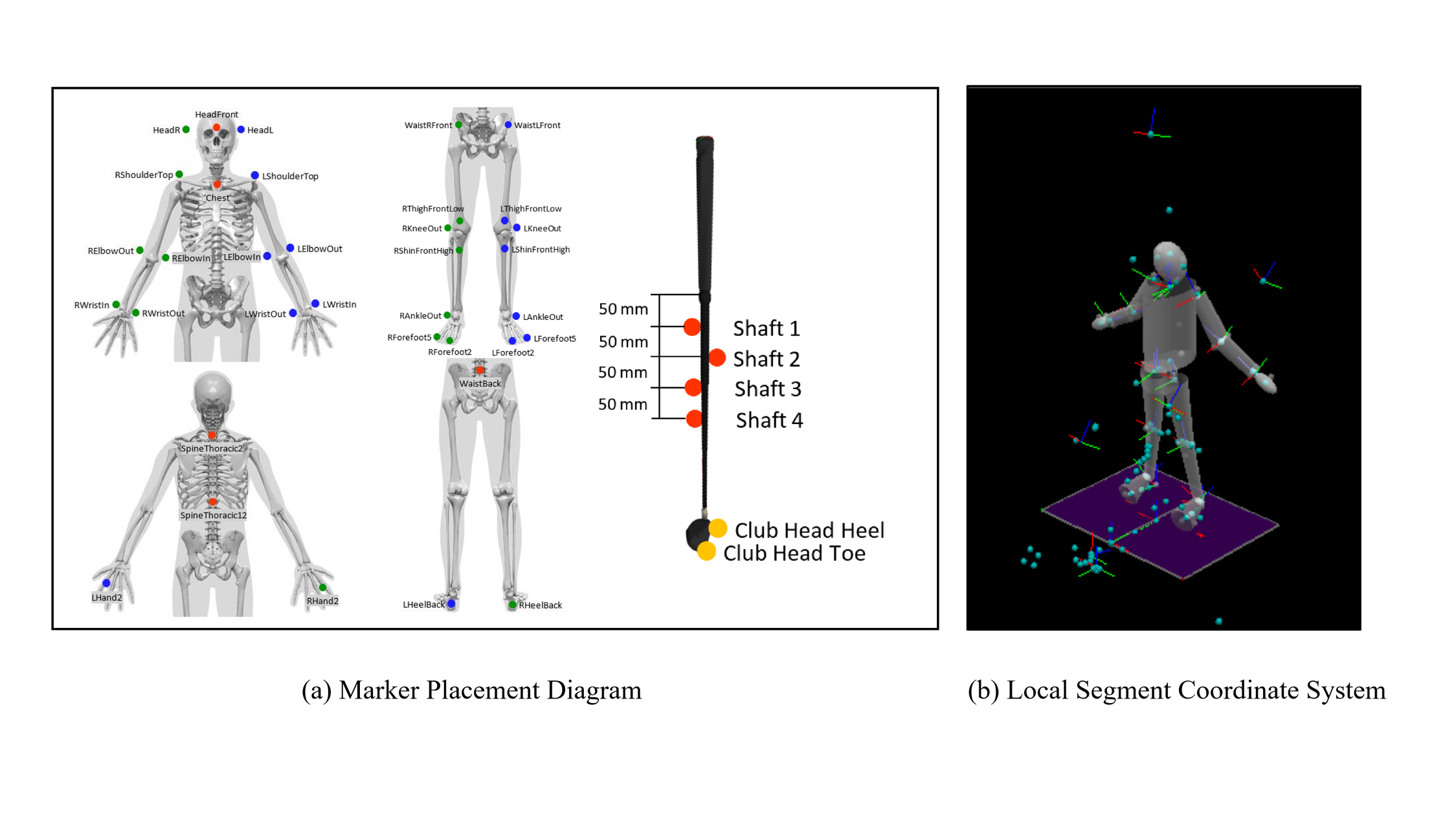

Supplement: S1 Fig — (TIF) [file pone.0331051.s001.tif]
